# Low-Intensity Extracorporeal Shock Wave Therapy Enhances Brain-Derived Neurotrophic Factor Expression through PERK/ATF4 Signaling Pathway

**DOI:** 10.3390/ijms18020433

**Published:** 2017-02-16

**Authors:** Bohan Wang, Hongxiu Ning, Amanda B. Reed-Maldonado, Jun Zhou, Yajun Ruan, Tie Zhou, Hsun Shuan Wang, Byung Seok Oh, Lia Banie, Guiting Lin, Tom F. Lue

**Affiliations:** Knuppe Molecular Urology Laboratory, Department of Urology, School of Medicine, University of California, San Francisco, CA 94143, USA; Bohan.Wang@ucsf.edu (B.W.); ninghongxiu@gmail.com (H.N.); Amanda.Reed-Maldonado@ucsf.edu (A.B.R.-M.); Jun.Zhou@ucsf.edu (J.Z.); Yajun.Ruan@ucsf.edu (Y.R.); Tie.Zhou@ucsf.edu (T.Z.); HsunShuan.Wang@ucsf.edu (H.S.W.); ByungSeok.Oh@ucsf.edu (B.S.O.); Lia.Banie@ucsf.edu (L.B.); glin@urology.ucsf.edu (G.L.)

**Keywords:** low-intensity extracorporeal shock wave treatment, brain-derived neurotrophic factor, PERK/ATF4 pathway, Schwann cells

## Abstract

Low-intensity extracorporeal shock wave therapy (Li-ESWT) is used in the treatment of erectile dysfunction, but its mechanisms are not well understood. Previously, we found that Li-ESWT increased the expression of brain-derived neurotrophic factor (BDNF). Here we assessed the underlying signaling pathways in Schwann cells in vitro and in penis tissue in vivo after nerve injury. The result indicated that BDNF were significantly increased by the Li-ESWT after nerve injury, as well as the expression of BDNF in Schwann cells (SCs, RT4-D6P2T) in vitro. Li-ESWT activated the protein kinase RNA-like endoplasmic reticulum (ER) kinase (PERK) pathway by increasing the phosphorylation levels of PERK and eukaryotic initiation factor 2a (eIF2α), and enhanced activating transcription factor 4 (ATF4) in an energy-dependent manner. In addition, GSK2656157—an inhibitor of PERK—effectively inhibited the effect of Li-ESWT on the phosphorylation of PERK, eIF2α, and the expression of ATF4. Furthermore, silencing ATF4 dramatically attenuated the effect of Li-ESWT on the expression of BDNF, but had no effect on hypoxia-inducible factor (HIF)1α or glial cell-derived neurotrophic factor (GDNF) in Schwann cells. In conclusion, our findings shed new light on the underlying mechanisms by which Li-ESWT may stimulate the expression of BDNF through activation of PERK/ATF4 signaling pathway. This information may help to refine the use of Li-ESWT to further improve its clinical efficacy.

## 1. Introduction

Although the phosphodiesterase inhibitors have positive effect on erectile dysfunction (ED), ED caused by nerve damage remains a challenge. The possibility to stimulate nerve growth could be expected to restore the erectile function. New insights to the mechanisms of cavernous nerves (CN) recovery may depend on novel neuromodulatory interventions. Brain-derived neurotrophic factor (BDNF) is a member of the mammalian family of neurotrophins. Because of its central role in neuronal development, maturation, and survival after injury, BDNF has been the focus of intense investigation [1]. BDNF is also being used as a treatment for various neurodegenerative diseases for its broad range of neurotrophic activities [2,3]. Previously, BDNF has been demonstrated to enhance the regeneration of neuronal nitric oxide synthase and recovery of erectile function [4,5]. BDNF is also involved in neurogenesis and neuroplasticity in the central nervous system. Growing evidence suggests that BDNF expression is changed in various neuropsychiatric disorders. There may be a correlation between low BDNF levels and bipolar disorder [6], the emergence of depression [7], and schizophrenia [8,9]. In addition, serum BDNF levels increase significantly in patients with severe mental illness during inpatient treatment. The increased levels are linked with clinical improvement [10]. Therefore, BDNF levels could be considered as a transdiagnostic marker for psychiatric disorder activity.

It is known that CN regeneration is a complex process in which Schwann cells (SCs) play a critical role through their de-differentiation, activation, proliferation, and re-differentiation, but how these events affect nerve regeneration is not clear. Our previous data established that BDNF promoted CN regeneration via SC-dependent Janus kinase (JAK)/signal transducer and activator of transcription (JAK/STAT) pathways [2,3]. BDNF can also play a role in SC-based myelination by recruiting partitioning-defective 3 (Par3) to the axon–glial interface [11]. Further evidence that SCs are a potentially effective therapeutic target for improving penile nerve regeneration comes from the positive effects of glial cell-derived neurotrophic factor (GDNF) application and SC transplantation [12]. However, the mechanisms involved in these effects have not been elucidated.

Low-intensity extracorporeal shock wave therapy (Li-ESWT) has been used for years to treat musculoskeletal disorders [13]. Recently, the application of this therapy has been expanded to address ischemic heart disease [14] and vasculogenic ED [15], but there are few reports concerning the effects Li-ESWT have on nerve fibers. Here are the several publications describing re-innervation of tissues after stimulation by this treatment. For example, Ohtori et al. reported Li-ESWT-induced re-innervation by sensory fibers in 2001 [16], and another Japanese group found that Li-ESWT stimulated the expression of growth-associated protein-43 (GAP-43) in rat dorsal root ganglia (DRG) in 2006 [17]. Li-ESWT has also been reported to stimulate DRG cells to express activating transcription factor 3 (ATF3), which promotes neurite outgrowth from the ganglion when the peripheral nerve is injured [17]. Our team recently reported that Li-ESWT improves diabetic ED in an animal model by promoting nerve regeneration [18] (a finding confirmed by another group [19]), as well as in a pelvic neurovascular injury model [20]. We now propose the application of Li-ESWT as a novel and non-invasive therapy to focally promote SC proliferation and differentiation to improve CN regeneration.

The endoplasmic reticulum (ER) is essential for cellular activities. In order to retain normal ER functions, the unfolded protein response (UPR) is an elementary response for accumulation of unfolded proteins. Protein kinase RNA-like ER kinase (PERK) is an important branch which is responsible for the attenuation of the overload of misfolded proteins, consequently attenuating ER stress [21]. PERK phosphorylates activate the α subunit of eukaryotic initiation factor 2 (eIF2α), which can subsequently allow the translation of UPR-dependent genes, such as ATF4. ATF4 stimulates the expression of ER stress target genes, including DNA-damage-inducible 34 (GADD34) and C/EBP-homologous protein (CHOP) [22].

With the purpose of further characterizing the effects and the mechanism of Li-ESWT on BDNF, the aims of this study can be outlined as follows: (1) to evaluate the effect of Li-ESWT on the expression of BDNF both in penile tissue and in SCs; (2) To evaluate the effect of Li-ESWT on PERK/ATF4 pathway in SCs; (3) To evaluate the function of the PERK/ATF4 pathway on the expression of BDNF.

## 2. Results

### 2.1. Expression Levels of BDNF in Penis Were Significantly Increased by Li-ESWT after Nerve Injury

**Figure 1:**
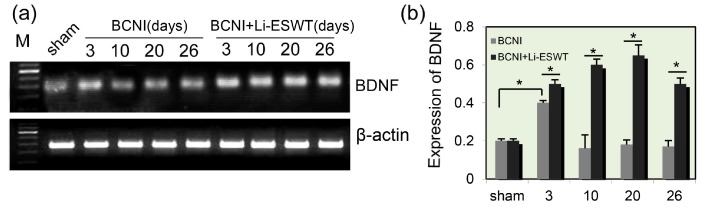
Expression levels of brain-derived neurotrophic factor (BDNF) in penis were significantly increased by the low-intensity extracorporeal shock wave therapy (Li-ESWT) after nerve injury. Bilateral cavernous nerve crush injury (BCNI) rats were treated with Li-ESWT (0.06 mJ/mm^2^, 3 Hz, 500 pulses) twice a week. (**a**) BDNF expression levels in penile tissues in control and Li-ESWT groups were measured by reverse transcription-polymerase chain reaction (RT-PCR) at 3, 10, 20, and 26 days (*n* = 6); (**b**) Compared to the control group, the expression levels of BDNF in penis was significantly increased at 3, 10, 20, and 26 days (*p* < 0.05) by the usage of Li-ESWT, and kept at a stable level until up to 26 days after the nerve injury. M: DNA molecular weight marker.

Bilateral cavernous nerve crush injury (BCNI) rats were treated with Li-ESWT (0.06 mJ/mm^2^, 3 Hz, 500 pulses) twice a week. BDNF expression levels in penile tissues were measured by reverse transcription-polymerase chain reaction (RT-PCR) at 3, 10, 20, and 26 days. mRNA levels of BDNF were significantly elevated after BCNI at 3 days. Compared to the sham group, expression of BDNF was significantly increased 3 days after nerve injury. In the control (BCNI without Li-ESWT) group, the BDNF expression decreased sharply, reaching the lowest level at 10 days post injury, increasing slightly at 20 days, and stabilizing at 26 days. Compared to the control group, the expression levels of BDNF in penis in the Li-ESWT group was significantly increased by the use of Li-ESWT, and kept at a stable level up to 26 days after the nerve injury (Figure 1a,b, *p* < 0.05).

### 2.2. Li-ESWT Increased Expression of BDNF in RT4-D6P2T Schwann Cells by Activating PERK/ATF4

**Figure 2:**
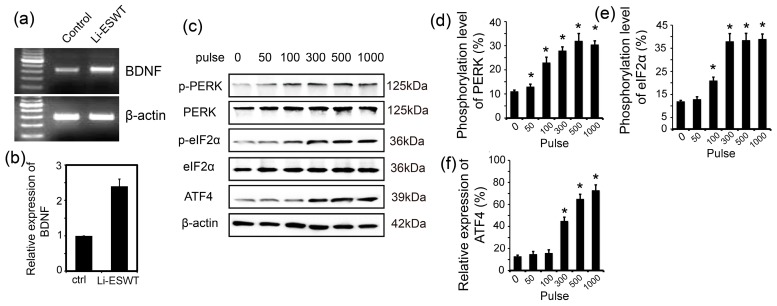
Li-ESWT treatment enhanced BDNF expression in RT4-D6P2T cells and activated intracellular signaling in a pulse-dependent fashion. (**a**) BDNF expression levels in SCs cells in control and Li-ESWT groups (*n* = 3 in triplicates); (**b**) BDNF expression was higher in Li-ESWT groups (*p* < 0.05); (**c**) Protein levels of p-PERK (phosphorylated protein kinase RNA-like endoplasmic reticulum (ER) kinase), PERK, p-eIF2α (phosphorylated eukaryotic initiation factor 2 α), eIF2α, ATF4, and β-actin at different pulses (0, 50, 100, 300, 500, and 1000) (*n* = 3 in triplicates); (**d**) Phosphorylation level of PERK increased significantly at pulses 50, 100, 300, 500, and 1000 (* *p* < 0.05). Intensity ratios depicted in corresponding bar graphs were calculated using phosphorylated and total protein expression; (**e**) Phosphorylation level of eIF2α increased significantly at pulses 100, 300, 500, and 1000 (* *p* < 0.05). Intensity ratios depicted in the corresponding bar graphs were calculated using phosphorylated (p-) and total protein expression; (**f**) Expression level of ATF4 increased significantly at pulses (300, 500, and 1000, * *p* < 0.05). Intensity ratios depicted in the corresponding bar graphs were calculated using ATF4 and β-actin expression.

Nerve regeneration is a complex process in which Schwann cells (SCs) play a critical role through their de-differentiation, activation, proliferation, and re-differentiation. Li-ESWT increased the expression of BDNF in RT4-D6P2T significantly (*p* < 0.05, Figure 2a,b) in vitro. Li-ESWT activated the PERK pathway by increasing the phosphorylation level of PERK and eIF2α, and enhanced ATF4 expression in an energy-dependent manner.

The expression of PERK and eIF2α were consistent at each energy pulse. In contrast, the phosphorylation level of p-PERK increased at 50 pulses and peaked at 500 pulses (*p* < 0.05, Figure 2c,d). p-eIF2α responded later than p-PERK, as phosphorylation was significantly enhanced at 100 pulses and peaked at 1000 pulses (*p* < 0.05, Figure 2e). Activation of ATF4 increased slightly at 50 pulses, increased significantly at 300 pulses, and reached a peak at 1000 pulses (*p* < 0.05, Figure 2c,f).

### 2.3. GSK2656157 Effectively Inhibited the Effect of Li-ESWT on the Phosphorylation of PERK and eIF2α and the Expression of ATF4 in RT4-D6P2T

**Figure 3:**
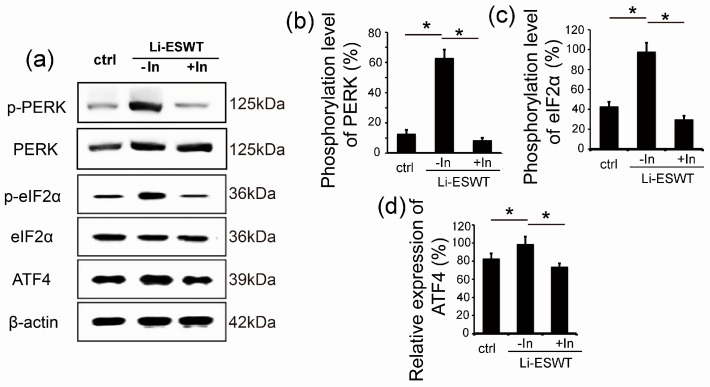
GSK2656157 effectively inhibited the phosphorylation of PERK and eIF2α and the expression of ATF4 in RT4-D6P2T. (**a**) Expression of p-PERK, PERK, p-eIF2α, eIF2α, and ATF4 with usage of Li-ESWT and PERK inhibitor (In) GSK2656157 (*n* = 3 in triplicates); (**b**) The phosphorylation level of PERK increased significantly with Li-ESWT, and could be blocked by GSK2656157 (* *p* < 0.05). Intensity ratios depicted in the corresponding bar graphs were calculated using phosphorylated (p-) and total protein expression; (**c**) Phosphorylation level of eIF2α increased significantly with Li-ESWT, and could be blocked by GSK2656157 (* *p* < 0.05). Intensity ratios depicted in corresponding bar graphs were calculated using phosphorylated (p-) and total protein expression; (**d**) Expression of ATF4 increased significantly with Li-ESWT, and could be blocked by GSK2656157 (* *p* < 0.05). Intensity ratios depicted in the corresponding bar graphs were calculated using ATF4 and β-actin expression.

Stimulation in the endoplasmic reticulum results in ER stress, which in turn activates a cellular pathway known as the unfolded protein reaction. PERK exists in the ER and is one of the three UPR-initiating proteins. In this study, Li-ESWT was used to simulate the expression of p-PERK (phosphorylated PERK), p-eIF2α, and ATF4 (the downstream target gene) (*p* < 0.05, Figure 3a). GSK2656157—a known PERK inhibitor—was used, and it was found that expression of phosphorylation of PERK, eIF2α, and ATF4 were attenuated sharply (*p* < 0.05, Figure 3a–d).

### 2.4. The Silencing of ATF4 Attenuated the Effect of Li-ESWT on the Expression of BDNF Dramatically, but Had No Effect on HIF1α and GDNF in RT4-D6P2T

**Figure 4:**
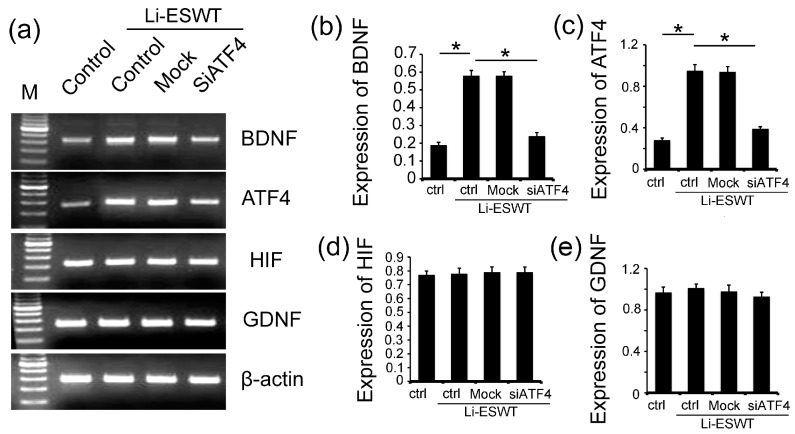
Silencing of ATF4 with siRNA in RT4-D6P2T cells in vitro. (**a**) The expression of BDNF, ATF4, hypoxia-inducible factor (HIF)1α, and glial cell-derived neurotrophic factor (GDNF) with siRNA and Li-ESWT (*n* = 3 in triplicates); (**b**) Silencing of ATF4 eliminated the effect of Li-ESWT on the expression of BDNF (*p* < 0.05); (**c**) Silencing of ATF4 eliminated the effect of Li-ESWT on the expression of ATF4 (* *p* < 0.05); (**d**) siATF4 and Li-ESWT had no effect on HIF1α (* *p* > 0.05); (**e**) siATF4 and Li-ESWT had no effect on GDNF (* *p* > 0.05).

In order to study the effect of ATF4 on BDNF, we used siRNA to silent the expression of ATF4 in RT4-D6P2T. The BDNF expression level was significantly increased by Li-ESWT in vitro (Figure 4a,b, *p* < 0.05). However, this increment could be eliminated by the use of siATF4. The expression of ATF4 had the same pattern as BDNF (Figure 4a,c, *p* < 0.05). Hypoxia-inducible factors (HIFs) are transcription factors that respond to decreases in available oxygen in the cellular environment, or hypoxia. Glial cell-derived neurotrophic factor (GDNF) is a protein that potently promotes the survival of many types of neurons. For the expression of HIF1α and GDNF, no differences were observed with the treatment of Li-ESWT and siATF4 (Figure 4a,d,e, *p* > 0.05).

## 3. Discussion

Neurotrophic factors enhance the process of nerve regeneration and functional recovery in animal models. One member of the neurotrophic factor—BDNF—has been shown to upregulate the function of several neurons in the peripheral nervous system. Hiltunen et al. reported the first case of retrograde transport of radio-labeled BDNF injected into the corpora cavernosa [23]. After injection of ^125^I-BDNF, radiography presented clustered silver grains in the major pelvic ganglion (MPG). BDNF was established as a potential candidate for neurogenic impotence because it could transport from axon terminals to the neuronal cell body. BDNF helps to support the survival of neurons and encourage the growth and differentiation of new neurons. BDNF has two major receptors: the pan-NT receptor p75 (p75^NTR^) and the tropomyosin-related receptor kinase B (TrkB) [24]. p75^NTR^ has many functions, such as differentiation, cell survival, and signal of cell death [25]. Binding of BDNF to TrkB triggers the activation of downstream signaling pathways, such as the RAS/mitogen-activated protein kinase (MAPK), RAP/MAPK, phosphoinositide 3-kinase (PI3K)/Protein Kinase B (AKT), and Phosphoinositide phospholipase C (PLCγ) pathways [24]. These pathways result in cell survival, differentiation, synapse formation, and plasticity. The BDNF/TrkB signaling pathway seems to have functions with tumor progression, such as peritoneal carcinomatosis [26], bladder cancer [27], and non-small cell lung cancer [28]. p75^NTR^ has been reported to be associated with tumor survival and resistance to drugs in breast tumor [29]. In our previous work, we demonstrated that BDNF promoted neurite growth in the MPG of the rat via the JAK/STAT pathway [3]. We also showed that the expression of penile BDNF was unregulated after cavernous nerve transection, and that the JAK/STAT signaling pathway was upregulated in the major pelvic ganglion [1]. In the current study’s control group, we found that expression of BDNF was significantly increased 3 days after nerve injury. Afterwards, the expression of BDNF decreased sharply, and reached basal level at 10 days post injury. With the use of Li-ESWT, the expression levels of BDNF in the penis increased and then remained at a stable level up to 26 days after the nerve injury. In brief, Li-ESWT could significantly promote the expression of BDNF in vivo.

In the first few days following the injury of myelinated nerve fibers, axonal degeneration proceeds distally from the injury site, and the surrounding myelin sheaths disintegrate. Macrophages soon invade and phagocytize the debris, while SCs de-differentiate and divide within their basal laminae, producing cordons of cells known as Bands of Buengner. During de-differentiation (which is crucial for nerve regeneration after injury), SCs retrogress to a primitive form and cease to express myelin-related genes [30]. In vitro study has shown that SCs can be successfully modified with the use of retroviral vectors to overexpress neurotrophic factors such as BDNF [31]. As such, we used SCs for our in vitro experiments to study the effect of Li-ESWT on the BDNF. We found that at the energy level of 0.01 mJ/mm^2^, 3 Hz, 300 pulses, Li-ESWT could enhance the expression of BDNF. Therefore, we could verify the effect of Li-ESWT on BDNF both in vivo and in vitro.

Li-ESWT has been applied in clinical treatment recently [20]. The clinical outcome of Li-ESWT is closely related to the energy flux density (EFD) [32]. The setup EFD varied from 0.09 to 0.25 mJ/mm^2^, and the pulses of shock wave varied from 1500 to 5000 among studies included in our previous works. Different energy densities were investigated for the regenerative purposes of Li-ESWT. Abe et al. revealed that an energy level of 0.1 mJ/mm^2^ had a good anti-inflammatory effect for a rat model of acute myocardial infarction [33]. For skin burns, Goertz et al. showed that at 0.04 mJ/mm^2^ could accelerate angiogenesis [34]. Tara et al. reported that 0.11–0.21 mJ/mm^2^ could improve therapeutic angiogenesis for human ischemic tissues [35]. Ioppolo et al. [36] found positive effects of using Li-ESWT at 0.3 mJ/mm^2^ as a treatment for musculoskeletal disorders, with a success rate ranging from 65% to 91%. In the current study, we used an energy density of 0.06 mJ/mm^2^, 3 Hz, 500 pulses to treat the penile tissue. In the in vitro study, 0.01 mJ/mm^2^, 3 Hz, 300 pulses was applied to treat the SCs. In our experience, the energy level applied is different for tissue and for cells. Although the energy density employed in this study was a little bit low compared to the previous studies, the outcomes for this EFD presented encouraging results. Therefore, our subsequent studies adopted this EFD.

In our previous study, we established that BDNF promoted nerve regeneration by activating the JAK/STAT pathway in Schwann cells [37]. Weihs et al. demonstrated that Li-ESWT triggered the release of cellular ATP, which subsequently enhanced proliferation in vitro and in vivo via Erk1/2 signaling pathway [38]. In our current study, we evaluated the effect of Li-ESWT on the PERK/ATF4 pathway, one mechanistic branch of the unfolded protein response (UPR).

The majority of proteins first enter the ER, where they fold and assemble. From the ER, only properly folded and assembled proteins develop to the cell surface. The ER responds to the burden of unfolded proteins by activating intracellular signal pathways, collectively called the UPR. In order to maintain homeostasis in the ER or to induce apoptosis, at least three mechanistic branches of the UPR regulate the expression of many genes. Each branch is defined by a class of transmembrane ER-resident signaling components: activating protein kinase RNA-like ER kinase (PERK), inositol requiring protein-1 (IRE1), and transcription factor-6 (ATF6) [39]. PERK is an important branch that is responsible for the attenuation of the overload of misfolded proteins, therefore alleviating ER stress. In our study, we evaluated whether Li-ESWT could promote the expression of p-PERK, p-eIF2α, and ATF4. Our results indicate that the PERK/ATF4 pathway was activated by Li-ESWT. Furthermore, different numbers of pulses (0, 50, 100, 300, 500, and 1000) were applied in our current study. We found that Li-ESWT activated the PERK pathway by increasing the phosphorylation level of PERK and eIF2α, and enhanced ATF4 expression in an energy-dependent manner. In addition, GSK2656157 attenuated the effect of Li-ESWT on the phosphorylation of PERK, eIF2α, and the expression of downstream target gene ATF4 effectively. GSK2656157 is an inhibitor of protein kinase R (PKR)-like endoplasmic reticulum kinase [40], which was selected for advancement to preclinical development. We found that by silencing ATF4, the expression of BDNF dramatically decreased, but HIF1α and GDNF were not affected.

BDNF and GDNF are important for the maintenance, survival, and regeneration of specific neuronal populations. The depletion of these neurotrophic factors has been associated with neurodegenerative diseases, such as Huntington’s disease, Parkinson’s disease, and Alzheimer’s disease. Preclinical results suggested that BDNF might promise to be an effective treatment [41].

Hypoxia means the state of low oxygen concentration. The HIF1α signaling cascade mediates the effects of hypoxia on the cell. In general, HIF1α plays a central role in the regulation of human metabolism [42]. In our study, we found that Li-ESWT increased BDNF significantly but had no effect on HIF1α and GDNF, which showed that BDNF could be the specific target for Li-ESWT and the PERK/ATF4 pathway.

## 4. Materials and Methods

### 4.1. Study Design

A total of 54 male Sprague Dawley rats (11 weeks old) were obtained from Charles River Laboratories (Wilmington, MA, USA), and were randomly divided into three groups (sham, control, and Li-ESWT treatment group). One group underwent sham surgery. The remaining animals underwent BCNI [43]. At different time points (3, 10, 20, and 26 days), penile tissues were excised for assessment of the mechanism. All procedures were approved by the Institutional Animal Care and Use Committee of University of California, San Francisco (Approval number: AN101658, July 2015. San Francisco, CA, USA).

### 4.2. BCNI Injury Model Establishment

Surgery was performed under 2% isoflurane anesthesia with the animal placed on a heating pad. A lower abdominal midline incision was used to expose the prostate gland. The major pelvic ganglion (MPG) and cavernous nerve (CN) were exposed on either side of the prostate. In sham group, the abdomen was then closed. In the treatment and control groups, standardized bilateral CN crush injury was performed by application of a designated needle driver 5-mm distant to the origin of the CN at the MPG for the duration of 2 min. The abdomen was then closed in two layers [43].

### 4.3. Low-Intensity Extracorporeal Shock Wave Treatment

For the in vivo experiment, Li-ESWT therapy was started 48 h post-operatively. Shock wave was delivered to the pelvic region with a special probe that was attached to a compact electromagnetic unit with an unfocused shockwave source (LiteMed, Taipei, Taiwan). Under anesthesia, each rat was placed in the supine position with its lower abdomen shaved and the preputial skin reduced. Standard commercial ultrasound gel (Aquasonic, Parker Laboratories, Inc., Fairfield, NJ, USA) was applied between the probe and the skin of pelvic region for optimal coupling. Energy of 0.06 mJ/mm^2^, 3 Hz, and 500 pulses was applied.

### 4.4. Rat Schwann Cell Treatment

RT4-D6P2T (rat Schwann) cells were used to check the effect of Li-ESWT on the PERK/ATF4 pathway. In brief, the cells were treated with or without Li-ESWT at different energy levels of 0.01 mJ/mm^2^, 3 Hz, (0, 50, 100, 300, 500, and 1000) pulses. After Li-ESWT treatment, the cells were incubated for another 8 h and preceded to Western Blot (WB) and RT-PCR to detect the activation of PERK pathway by checking p-eIF2α and ATF4. Afterwards, the cells were treated with or without PERK inhibitor (1 μM GSK2656157) for 1 h, and then treated with or without Li-ESWT at the energy level of 0.01 mJ/mm^2^, 3 Hz, 300 pulses. After Li-ESWT treatment, the cells were incubated for another 8 h and preceded to WB and RT-PCR to detect the activation of the PERK pathway.

According to the protocol, transient transfection of siATF4 was performed using lipofectamine transfection reagent 2000 (Invitrogen, Carlsbad, CA, USA). Lipofectamine 2000 reagent was used in control cells only for mock transfection. RT4-D6P2T cells were seeded and cultured for 16 h until they reached 60%–70% confluence. The culture medium was replaced with antibiotic-free and serum-free medium at 1 h before transfection. The RT4-D6P2T cells were incubated with transfection mixtures containing 30 nM of siATF4 or mock for 5 h, and then the medium was replaced to full culture medium.

### 4.5. Reverse Transcription-Polymerase Chain Reaction

Total RNA (2.5 µg) was annealed to 0.4 µg of oligo-dT primer in a volume of 12 µL. Then, 4 µL of 5× buffer, 2 µL of 0.1 mol/L Dithiothreitol (DTT), 1 µL of 10 mmol/L dNTP, and 1 µL of SuperScript reverse transcriptase (Invitrogen, La Jolla, CA, USA) were added to bring the final volume to 20 µL. After 1 h of incubation at 42 °C, the mixture was incubated at 70 °C for 10 min to inactivate the reverse transcriptase. Then, 80 µL of Tris-EDTA (TE) buffer was added to make a 5× diluted complementary DNA library, from which 1 µL was used for RT-PCR. The cycling program was set for 35 cycles of 94 °C for 10 s, 55 °C for 10 s, and 72 °C for 10 s, followed by one cycle of 72 °C for 5 min. The PCR products were electrophoresed in 1.5% agarose gels, visualized by ultraviolet fluorescence, and recorded by a digital camera. Data were analyzed by ChemiImager-4000 software (Version 4.04, Alpha Innotech Corporation, San Leandro, CA, USA). Primer sequences are presented in Table 1.

### 4.6. Western Blotting

RT4-D6P2T cells were homogenized in lysis buffer containing 1% IGEPAL, 0.5% sodium deoxycholate, 0.1% SDS, 0.1 mM Na_3_VO_4_, aprotinin (10 µg/mL), and leupeptin (10 µg/mL). The homogenate was centrifuged at 14,000× *g* for 10 min, and the supernatant was recovered as protein sample, which was measured for protein concentration by the BCA method (Pierce Chemical Company, Rockford, IL, USA) and analyzed by Western blotting as previously described [37]. The primary antibodies used in these experiments were phospho- and total PERK, phospho- and total eIF2α, ATF4, and β-actin (Cell Signaling Technology, Beverly, MA, USA). GSK2656157—a PERK inhibitor—was obtained from Calbiochem (San Diego, CA, USA). The resulting image was analyzed with ChemiImager 4000 to determine the integrated density value (IDV) of each protein band.

### 4.7. Statistical Analyses

Means ± S.D are given in all graphs. *p*-Values were calculated using one way ANOVA followed by either Dunnett’s or Boneferroni’s multiple comparison tests. For single comparisons, we used a Student’s *t-*test. *p* < 0.05 was considered as a significant difference.

## 5. Conclusions

In conclusion, Li-ESWT may stimulate the expression of BDNF through the activation of the PERK/ATF4 signaling pathway. Although more studies are still required to further define these processes, we think that our research may help to refine the use of Li-ESWT to further improve its clinical efficacy.

## Figures and Tables

**Table 1:** RT-PCR primers and siRNA (RT-PCR = reverse transcription-polymerase chain reaction).

Gene	Sequence of Primers (5′–3′)
*BDNF*	Forward: GATGCTCAGCAGTCAAGTGCCTTT
	Reverse: AGAAAGAGCAGAGGAGGCTCCAAA
*ATF4*	Forward: ATGGCTGGCTATGGATGG
	Reverse: GGGAAGAGGCTGCAAGAA
*HIF1α*	Forward: CATCTCCACCTTCTACCC
	Reverse: TCCAAGAAAGCGACATAG
*GDNF*	Forward: CCAGAGAATTCCAGAGGGAAAG
	Reverse: TCAGATACATCCACACCGTTTAG
*β-Actin*	Forward: CTACAATGAGCTGCGTGTG
	Reverse: AATGTCACGCACGATTTCCC
*siATF4*	Forward: GCCATCTCCCAGAAAGTGTAATA
	Reverse: GTCATAAGGTTTGGGTCGAGAA
